# Supplementary material for: Saccharomyces boulardii (CNCM I-745) improves intestinal damage in sepsis by remodeling intestinal flora structure
Source: Front Cell Infect Microbiol. 2025 Jul 31;15:1602792. doi: 10.3389/fcimb.2025.1602792 (PMC12350329; doi:10.3389/fcimb.2025.1602792)
Supplement: Supplementary file 1 [file DataSheet1.zip › summary/summary/8_Advanced_analyse/2_PICRUSt2_result/METACYC/Group.CW0_vs_AW0/PICRUSt2_METACYC_Group.CW0_vs_AW0_diff.pdf]

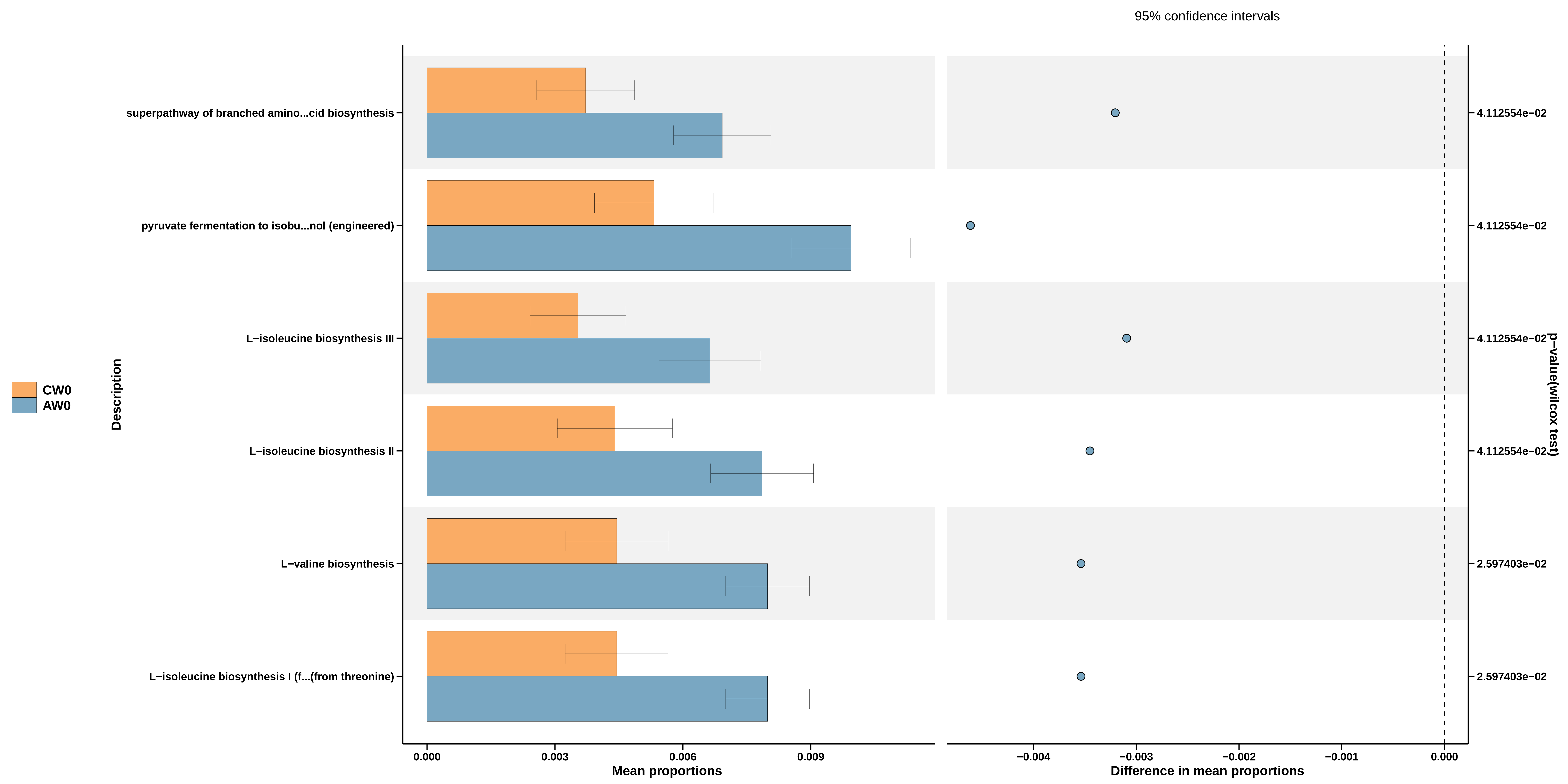
95% confidence intervals
superpathway of branched amino...cid biosynthesis
pyruvate fermentation to isobu...nol (engineered)
L−isoleucine biosynthesis III
L−isoleucine biosynthesis II
L−valine biosynthesis
L−isoleucine biosynthesis I (f...(from threonine)
Description
CW0
AW0
0.000
0.003
0.006
0.009
Mean proportions
−0.004
−0.003
−0.002
−0.001
0.000
Difference in mean proportions
4.112554e−02
4.112554e−02
4.112554e−02
4.112554e−02
2.597403e−02
2.597403e−02
p−value(wilcox test)
